# Correlation coefficient-directed label-free characterization of native proteins by surface-enhanced Raman spectroscopy†

**DOI:** 10.1039/d2sc04775f

**Published:** 2022-10-31

**Authors:** Ping-Shi Wang, Hao Ma, Sen Yan, Xinyu Lu, Hui Tang, Xiao-Han Xi, Xiao-Hui Peng, Yajun Huang, Yi-Fan Bao, Mao-Feng Cao, Huimeng Wang, Jinglin Huang, Guokun Liu, Xiang Wang, Bin Ren

**Affiliations:** State Key Laboratory of Physical Chemistry of Solid Surfaces, Collaborative Innovation Center of Chemistry for Energy Materials (i-ChEM), Innovation Laboratory for Sciences and Technologies of Energy Materials of Fujian Province (IKKEM), Department of Chemistry, College of Chemistry and Chemical Engineering, Xiamen University Xiamen 361005 China oaham@xmu.edu.cn wangxiang@xmu.edu.cn bren@xmu.edu.cn; Laser Fusion Research Center, China Academy of Engineering Physics Mianyang 621900 China; State Key Laboratory of Marine Environmental Science, College of the Environment and Ecology, Xiamen University Xiamen 361005 China

## Abstract

Investigation of proteins in their native state is the core of proteomics towards better understanding of their structures and functions. Surface-enhanced Raman spectroscopy (SERS) has shown its unique advantages in protein characterization with fingerprint information and high sensitivity, which makes it a promising tool for proteomics. It is still challenging to obtain SERS spectra of proteins in the native state and evaluate the native degree. Here, we constructed 3D physiological hotspots for a label-free dynamic SERS characterization of a native protein with iodide-modified 140 nm Au nanoparticles. We further introduced the correlation coefficient to quantitatively evaluate the variation of the native degree, whose quantitative nature allows us to explicitly investigate the Hofmeister effect on the protein structure. We realized the classification of a protein of SARS-CoV-2 variants in 15 min, which has not been achieved before. This study offers an effective tool for tracking the dynamic structure of proteins and biomedical research.

## Abstract 2

Iodide modified Au nanoparticles combined with correlation coefficient allows label-free SERS characterization of proteins in native state and analysis of Hofmeister effect on protein structure, further enabling identification of protein variants.

## Graphical abstract

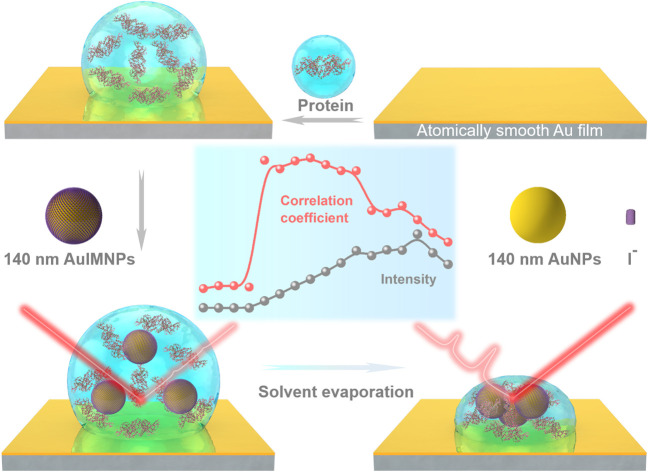

## Introduction

Proteins are the material basis of life activities and have to be in their native state to precisely perform the specific functions, such as signaling,^1^ transporting,^2,3^ and catalyzing.^4,5^ The in-depth investigation of proteins in their native state is the core of current proteomics toward a better understanding of their structures and functions. One of the important aspects of proteomics is to precisely characterize the protein structure, which is tremendously important to find the diagnostic markers of disease, screen drug targets and so on.^6^ Conventional approaches, including mass spectroscopy,^7,8^ enzyme-linked immunosorbent assay,^9^ X-ray crystallography,^10,11^ and NMR,^12^ unveil the structural and/or functional information of proteins in different dimensions and have greatly facilitated the development of proteomics.^13,14^ It is desired to develop ultrasensitive, cost-effective, and simple characterization techniques to obtain the native and intrinsic protein structure in a physiological environment. Among different techniques, optical methods are one of the most powerful ways to achieve this goal.

Surface-enhanced Raman spectroscopy (SERS) has been accepted as a promising tool in proteomics, owing to its ability to provide fingerprint information in a noninvasive way and with single-molecule sensitivity.^15,16^ In 1980, Cotton *et al.* utilized surface-enhanced resonance Raman scattering for detection of cytochrome C (Cyt C) and myoglobin, which opened a door for SERS in protein detection.^17^ In fact, the SERS signals were dominated by the cofactors (*e.g.*, porphyrin and flavin adenine dinucleotide), due to their large Raman cross-section and resonance effect with proper incident light.^18^ Xu *et al.* also demonstrated the single-molecule detection of hemoglobin on silver nanoparticles (AgNPs), when hemoglobin is located in the gap of AgNP aggregates (*i.e.* hotspots).^19,20^ However, SERS spectra of a majority of proteins without cofactors are dominated by aromatic amino acid residues (phenylalanine, tryptophan, and tyrosine).^21^ It is challenging to detect other amino acids without aromatic rings. Zhao, Ozaki and their coworkers utilized acidified sulfate to trigger the aggregation of AgNPs with a protein. In this way, the protein could be trapped into the hotspots, resulting in a higher sensitivity.^22^ The synthesized nanoparticles are often capped with reductants or capping agents and negatively charged, which hinders their aggregation to form hotspots with biomolecules. To tackle this problem, Ramon *et al.* utilized spermine to modify the AgNPs with a positive charge for detecting DNA with high sensitivity.^23^ Our group introduced iodide-modified AgNPs (AgIMNPs) and utilized MgSO_4_ to trigger aggregation for highly sensitive protein detection. The strongly adsorbed monolayer iodides not only remove the reductants from the surface, but also prevent strong chemical interaction between the protein and metal surface.^24^ To date, this method has been extensively used for detecting different proteins as well as other biomolecules by introducing exogenous substances, such as aluminum ions,^25^ dichloromethane and CaCl_2_,^26^ to induce better aggregation.^27,28^

It can be found that all the above methods inevitably introduced exogenous substances (different ions or organics), which would possibly cause denaturation and trigger additional dominating docking positions of proteins. However, for a protein in the physiological environment, it is devoid of exogenous salts and organics and is fully hydrated. Furthermore, it undergoes Brownian motion and can be freely docked to the substrate.^29,30^ Therefore, it is highly important to construct hotspots under the physiological conditions (termed as physiological hotspots) without introducing exogenous species to obtain SERS spectra of the native protein. Recently, Yang *et al.* proposed dynamic SERS (D-SERS) to form controllable hotspots to capture different types of molecules by taking advantage of the evaporation of the solvent and nanocapillary pumping.^31,32^ This method shows high sensitivity and provides a new opportunity for constructing physiological hotspots.

Combining the advantages of iodide modification and D-SERS, we proposed a universal strategy for detecting proteins in their native state. We introduced iodide-modified gold nanoparticles (AuIMNPs) to construct dynamic 3D physiological hotspots on the atomically smooth hydrophobic gold surface (D-AuIMNPs) to obtain highly reproducible SERS spectra of proteins in the native state. We proposed to use the correlation coefficient between the SERS and normal Raman spectra as a criterion to evaluate the native degree of proteins indicated by the SERS spectra. The strategy not only enables us to explore the Hofmeister effect on the structure of the native protein by SERS, but also achieves the rapid and accurate identification of variant proteins of SARS-CoV-2, which has not been possible before.

## Results and discussion

**Fig. 1:**
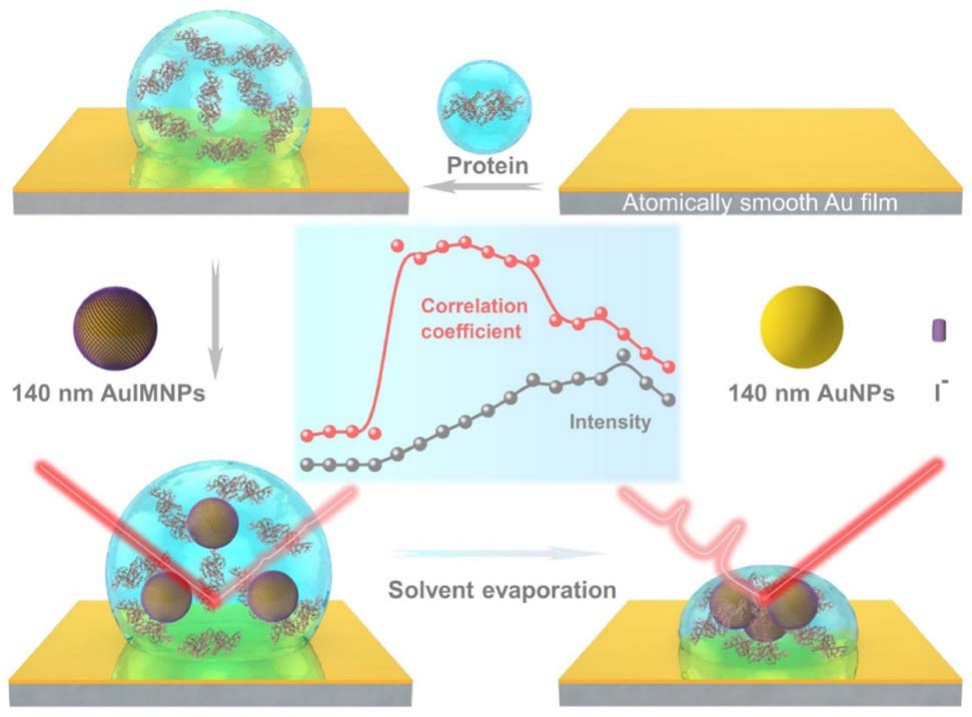
Schematic diagram of procedures for native protein characterization.

Fig. 1 illustrates the procedure for the native protein characterization and the models of the corresponding steps. We first introduced a hydrophobic template-stripped smooth gold film as the substrate. Note that the hydrophobic surface can confine the solution containing proteins and gold nanoparticles into a droplet. It should also feature low absorbance, high reflectance, and low interfering background. Thus, we utilized hydrophobic gold films as the support (see Fig. S1† for details). Then 2 μL of the protein solution (in the case of lysozyme, 200 μg mL^−1^) was dropped on the gold film, where the hydrophobic surface kept the solution as a droplet. To the droplet of the protein solution, 2 μL of 140 nm AuIMNPs (amounts to 4 × 10^7^) were added to form a mixture. During the evaporation of the solvent in the droplet, the gaps between nanoparticles shrank, which formed dynamic 3D physiological hotspots on the atomically smooth hydrophobic gold surface (D-AuIMNPs). This results in an increasing signal intensity. The signal intensity weakened when the droplet became dry. The correlation coefficient was calculated for all the SERS spectra relative to the normal Raman spectrum, as will be discussed in detail later. It is interesting to find that the trend of the correlation coefficient can well indicate the native state of the protein but the SERS intensity cannot. It should be pointed out that it is important to use 140 nm AuIMNPs for the following reasons: (1) iodide modification removes the reductants and impurities preventing a fluctuating interfering signal. The presence of a monolayer of atomic iodide prevents the strong interactions between the protein and metal surface, (2) AuNPs of 140 nm can provide much higher enhancement than that of small size under 785 nm excitation,^33^ as indicated by FDTD simulations (Fig. S2†), and (3) the synthesis of AuNPs is more controllable and stable (SEM, Fig. S3†) than the synthesis of AgNPs. When these advantages are combined with dynamic evaporation on the hydrophobic Au film without adding other agents, it allows the formation of dynamic 3D physiological hotspots to produce strong and highly reproducible SERS spectra of proteins in their native state.

**Fig. 2:**
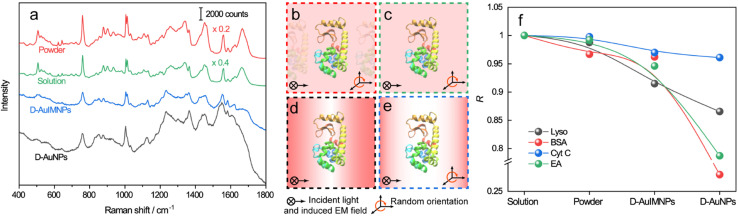
a) Normal Raman spectra of the powder and saturated aqueous solution of lysozyme, SERS spectrum of lysozyme with D-AuIMNPs, and SERS spectrum of AuNPs without KI modification. Models of lysozyme in different environments: (b) powder, (c) solution, (d) D-AuNPs, and (e) D-AuIMNPs. (f) Summaries of *R* change of lysozyme, BSA, Cyt C and EA in different environments.

The spectra of lysozyme in different environments are shown in Fig. 2a. It is obvious that the SERS spectrum obtained by D-AuIMNPs is highly similar to the normal Raman spectra in solution and powder forms, while the spectrum obtained by the D-AuNP substrate is very different from the others. A similar phenomenon has been reported in our previous work but is not fully understood.^24^ To clarify this phenomenon, we proposed models of lysozyme in different environments, as shown in Fig. 2b–e. For a protein in powder (Fig. 2b) and in solution (Fig. 2c) forms, the protein takes a random orientation in the uniform EM field generated by the excited light. The obtained spectrum is averaged over all orientations and every component of polarizability contributes to the final spectrum. This is the reason why their spectra are similar to each other. We regarded a protein in saturated aqueous solution (*ca.* 200 mg mL^−1^ for lysozyme) as the native state, but neglected the impact of crowding and the influence of protein–protein interactions in the model, which may contribute to the minor spectral difference from the spectra obtained in powders. For lysozyme on AuNPs (Fig. 2d), the spectral feature of SERS is obviously different from that of normal Raman spectra of a solid and solution. This is due to the strong interaction between the molecule and metal surface, which may lead to possible adsorption of a protein with a specific orientation and perturbation of the electronic structure of the molecule. Moreover, the surface selection rule and decay nature of the enhanced EM field in SERS will selectively enhance the groups close to the metal surface with a polarizability component parallel to the direction of the EM field. These two effects result in an intensity change and/or frequency shift, showing a very different SERS spectrum from a normal Raman spectrum. Furthermore, the SERS spectrum shows clear signals from reductants and impurities, which will interfere with the signal of lysozyme. These facts are responsible for bad reproducibility of SERS spectra of proteins. However, in the case of D-AuIMNPs (Fig. 2e), the obtained spectrum is similar to the normal Raman spectrum in solution but different from that on AuNPs. It may be attributed to the presence of the iodide monolayer, which prevents the direct interaction of lysozyme with the Au surface. In this way, lysozyme could move freely and take a random orientation. Furthermore, the gap size is estimated to be around 5 nm in the presence of an adsorbed iodide layer and protein, which results in a more uniform distribution of the EM field inside the gap (consistent with FDTD simulations, Fig. S2†) compared with that of NPs with a smaller size and in the absence of an iodide layer. This accounts for the phenomenon that its spectrum is similar to the normal Raman spectrum in solution, and the protein contributing to the spectrum can be regarded to be in its native state. As the hotspots are formed without adding other agents and under physiological conditions, we can term such types of hotspots in D-AuIMNPs 3D physiological hotspots.

The above analysis of the native state of proteins is more qualitative by comparing the spectral features in the Raman spectra. It would be more convincing if a more quantitative approach could be developed to describe the degree of similarity in the SERS spectra compared with the normal Raman spectrum of the native protein in solution. We thus proposed using the correlation coefficient (*R*) that is widely used in comparing the experimental and theoretical results in molecular spectroscopy.^34^
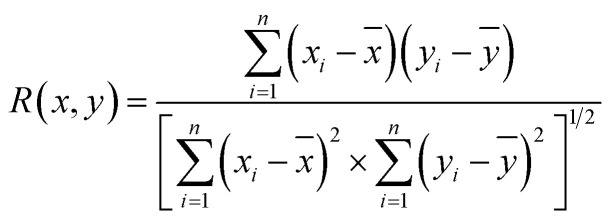
where *x*_*i*_ and *y*_*i*_ are the intensities of the SERS spectrum of lysozyme and the normal Raman spectrum of native lysozyme at *i* pixel, respectively. *x̄* and *ȳ* are the respective averaged intensities over all pixels (*n*). We use the normal Raman spectrum of lysozyme in solution as a representative of the native state, as it is closer to that of the physiological condition. *R* can be used to quantitatively evaluate the degree of natural conformation preserved based on the Raman spectra of lysozyme under different detection conditions and the result is shown in Fig. 2f. Naturally, the *R* value for the solution spectrum is 1. The spectrum obtained in powder revealed a high *R* of 0.99. The slight difference mainly comes from the weak interaction between proteins and a low hydrated state. As expected, the spectrum from D-AuIMNPs presents a high *R* of 0.91 owing to its 3D physiological hotspots whereas the spectrum obtained from AuNPs has an *R* of 0.86. The same strategy can be applied to evaluate the Raman spectra of bovine serum albumin (BSA, Fig. S4a†), Cyt C (Fig. S4b†) and egg albumin (EA, Fig. S4c†), and the *R* values for different proteins under different conditions are summarized in Fig. 2f. A similar trend to that of lysozyme verifies the universality of using *R* in determining the native state of proteins (>0.9).

**Fig. 3:**
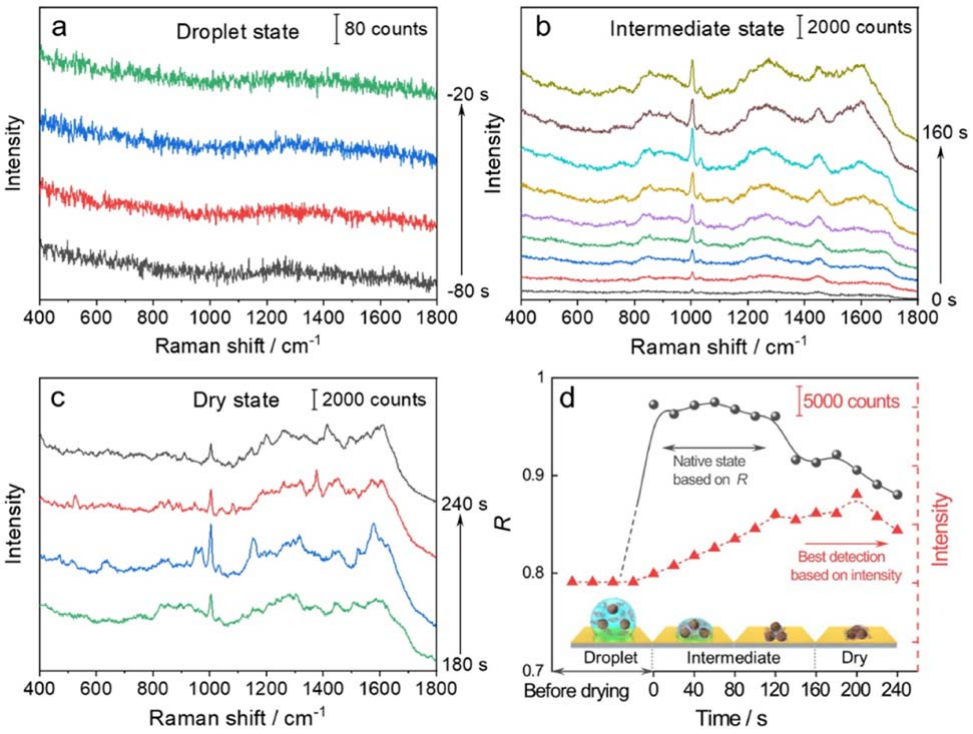
Time course of dynamic characterization of BSA and its corresponding SERS spectra in: the (a) droplet state, (b) intermediate state, and (c) dry state. (d) Correlation coefficients (*R*, solid line) and changes of the intensity of the peak at 1004 cm^−1^ (dotted line) during solvent evaporation. The *x*-axis represents the time corresponding to the specific stage: the droplet state (*t* < 0 s), intermediate state (0 to 160 s), and dry state (*t* > 160 s).

We then used the *R* value to monitor the dynamic changes in the native state of proteins with the evaporation of solvent and the formation of the 3D hotspots during SERS measurement. The whole process can be divided into four stages (Fig. 3): (1) droplet stage (Fig. 3a and S5a†). In this stage, the newly exfoliated hydrophobic gold film confines the mixture of AuIMNP sol and protein solution in a spherical form. The gap between AuIMNPs is too large to form aggregates and to give detectable signals. (2) Intermediate stage (Fig. 3b and S5b†). After vacuum-drying the droplet into a liquid film, the timing started. In the first 60 seconds of solvent evaporation, the adhesion force brings the nanoparticles closer, leading to the shrinking of the gap and increasing the strength of the EM field. As a result, we observed a gradual increase in the SERS intensity of the protein. Different from the gradual increase in intensity, the *R* value suddenly increases to above 0.97 for BSA and 0.88 for lysozyme, and reaches its maximum at about 60 s. Such a high coefficient is a result of the random orientation of the protein under conditions close to a physiological environment inside the quasi-uniform EM field of the hot spot. Therefore, the observed SERS spectra highly resemble the normal Raman spectrum of the native protein. After about 80 s, the three-phase interfaces of solid–liquid–air are formed, the AuIMNPs can be held more tightly, and the intensity keeps increasing.^31^ However, the *R* value starts to decrease because the proteins tend to stick to the AuIMNP surface and have less freedom to change their orientation under such a condition. For normal Raman and D-SERS measurements, the obtained spectrum is contributed by the molecules of all orientations. With the gap shrinking, the proteins have less freedom and tend to adsorb onto the surface. It implies that the final spectrum would be the averaged spectrum of several orientations (not fully sampled), resulting in a change in the spectrum and decrease in the *R* value. (3) Dry stage (Fig. 3c and S5c†). Starting from 140 s, the intensity of the protein is successively enhanced and reaches the maximum at 200 s, indicating that the gaps further shrank resulting in a more confined EM field and thus higher enhancement. In stark contrast to the intensity, the *R* continues decreasing and tends to be stabilized. It suggests that the natural structure of the protein is gradually broken and the dehydrated protein becomes the dominating species. Furthermore, the proteins tend to be adsorbed strongly on the AuIMNPs. These factors result in spectral features different from those of the intermediate state (more proteins are in native state) in this period. Note that such a dynamic change in the spectra during solvent evaporation cannot be observed by normal Raman spectroscopy due to its low sensitivity.

As summarized in Fig. 3d and S5d†, we conclude that the best period for obtaining SERS spectra of the native protein directed by the *R* is to acquire the spectra at 60 s (at the intermediate state) after vacuum drying, whereas the conventional intensity-based method points to the time period after 160 s, when the spectra have already lost the native intrinsic information of the protein. By combining the D-AuIMNPs with the *R*, we have not only elucidated the relationship between the intensity and native state in SERS characterization of proteins, but also monitored spectral changes when the protein enters physiological hotspots without introducing any exogenous species. D-AuIMNPs will enable the characterization of the dynamic structure of proteins *in situ* with controlled evaporation to keep the intermediate state.

**Fig. 4:**
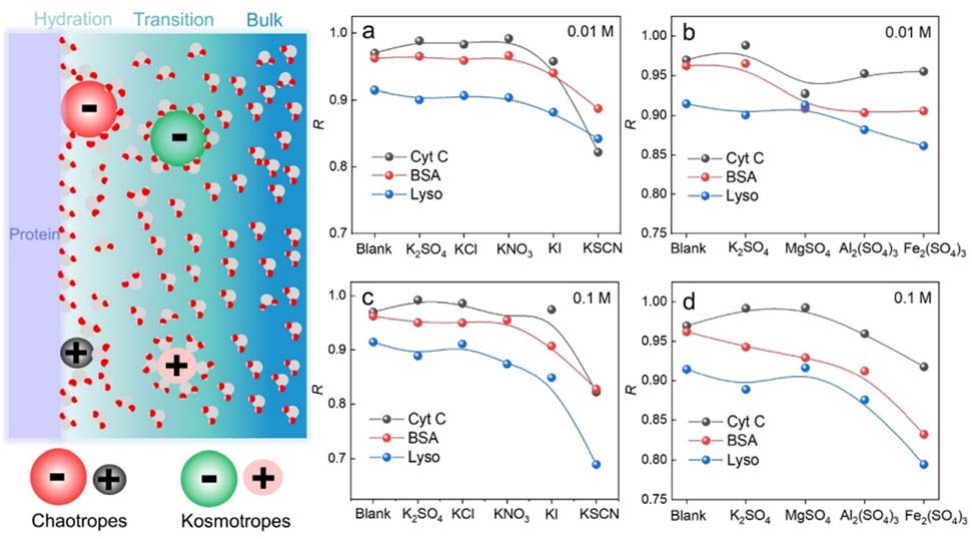
Schematic diagram of the protein interface and tendency of *R* change of proteins in different salt solutions: (a) different cations at 0.01 M, (b) different anions at 0.01 M, (c) different cations at 0.1 M, and (d) different anions at 0.1 M.

We have demonstrated that D-AuIMNPs can characterize the dynamic change of the protein in the native state according to the *R*, which enables us to monitor the perturbation of exogenous species on proteins. Here, we tried to investigate the effect of ions on the protein structure, not only because they are normal aggregation agents in SERS, but also due to their vital roles in physicochemical science. Desolvation induced by ions is the most important and common phenomenon in a protein solution, which has aroused a new research upsurge and has been confirmed to be involved in several important biological processes, such as protein folding and^35,36^ phase-separation.^37^ Basically, for a protein in the aqueous solution, the interface is usually divided into three layers: the hydration layer, transition layer and bulk solution,^38,39^ as shown in Fig. 4. When ions are added to the solution, they will exhibit different salting abilities, whose typical order is known as the Hofmeister series (cations, *i.e.* NH_4_^+^ > K^+^ > Na^+^ > Mg^2+^ > Ca^2+^ > Ba^2+^ > Al^3+^ > Fe^3+^, and anions, *i.e.*, SO_4_^2−^ > Cl^−^ > Br^−^ > NO_3_^−^ > I^−^ > SCN^−^). Many experimental methods, such as infrared spectroscopy, dynamic light scattering, NMR and nanopore are useful tools for studying the Hofmeister effects on protein behavior at high concentrations.^40–44^ It is of great significance to develop a quantitative method to describe and interpret the Hofmeister effect on the protein structure at low concentrations close to the physiological concentration (<0.1 M), which has rarely been reported. To this end, we sought to investigate the ion effect on the protein at low concentrations (0.01 M and 0.1 M) by D-AuIMNPs. As shown in Fig. 4, we showed the ion effect of different cations and anions on the protein at different concentrations by evaluating their *R*. Their spectra are shown in the ESI (Fig. S6–S8†). Generally, the ion effect of anions on proteins is greater than that of cations, and the effect of anions and cations is greater at a high concentration than that at a low concentration. As shown in Fig. 4a and c, the overall trend of anions is similar to the Hofmeister series. SO_4_^2−^, Cl^−^ and NO_3_^−^ are so-called kosmotropes and show the salting-out effect, which helps retain the native protein structure and results in a high *R*. I^−^ and SCN^−^ are chaotropes and show the salting-in effect, which would truncate the hydration layer and interact with the protein. They present a low *R*. As shown in Fig. S6,† the SERS spectra of lysozyme in the presence of I^−^ and SCN^−^ lose characteristic features of amide III and the intense peak at 1004 cm^−1^. It indicates that the negatively charged I^−^ and SCN^−^ will combine with the positively charged region of the protein, which leads to denaturation of the protein. It may even lead to a negative charge in the protein, which repels negatively charged AuIMNPs and makes it difficult for the protein to enter the hot spots even using D-SERS. In the case of cations, the overall trend is similar to the Hofmeister series as well, but is a little complex at a low concentration. It is to be noted that almost all metal cations are chaotropes, which interact with proteins by coordinating with amino acids.^45^ As indicated in Fig. 4b and d, the *R* decreases with the increase of the ionic charge. The widely used aggregation agents, like MgSO_4_ and Al_2_(SO_4_)_3_, denature the protein to varying degrees (Fig. S7†). Specifically, we can obtain the most intense spectra of BSA in the presence of Al_2_(SO_4_)_3_, which is consistent with the result by Guo *et al.*^25^ However, it has blurred many spectral features such as the characteristic peak of tyrosine at around 830 cm^−1^, which indicates possible denatured conformations existing in the solution. Taken together, we have quantitatively evaluated the effect of ions on the protein structure, and it can be found that different ions have different effects on different proteins. D-AuIMNPs completely avoid the use of these anions and cations, and can retain the native structure of proteins (*R* > 0.9).

**Fig. 5:**
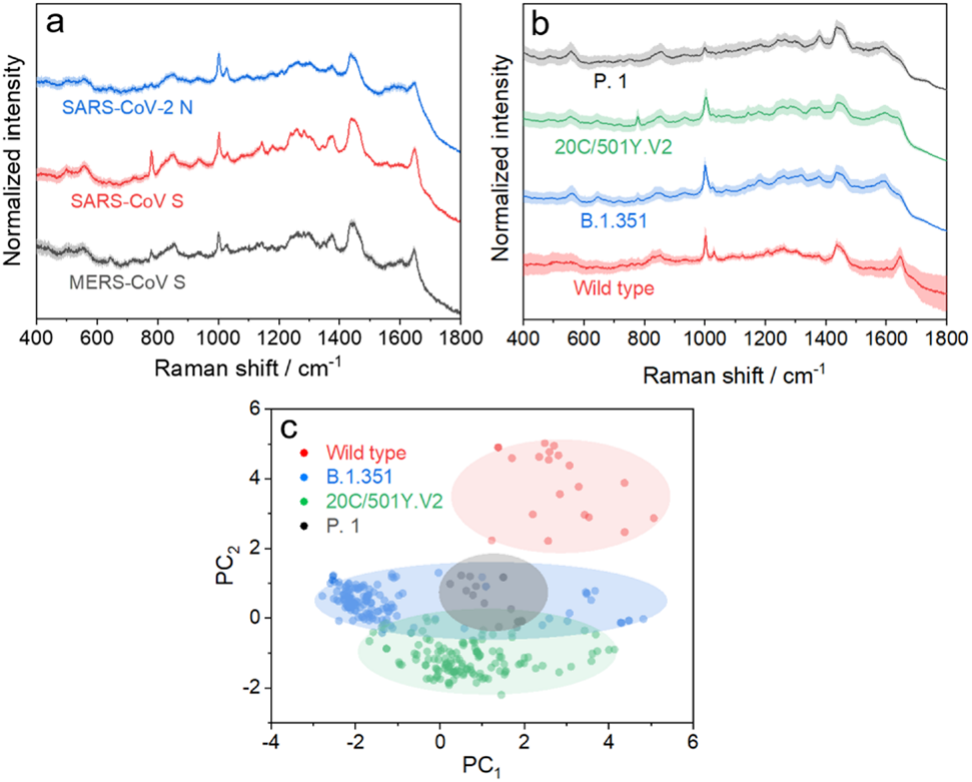
(a) SERS spectra of the SARS-CoV-2 N protein, SARS-CoV S protein, and MERS-CoV S protein. (b) SERS spectra of the S protein from wild type SARS-CoV-2 and its variants. (c) Radial visualization plot of the PC scores showing data from different variant proteins clustered together. Each dot corresponds to a SERS spectrum.

We further applied the above strategy to identify more challenging variants of SARS-CoV-2 (see Fig. S9 and S10† for details), which causes high infectivity, different symptoms, and evasion of humoral immunity.^46–48^ Mutation in emerging SARS-CoV-2 variants mostly occurs in the RNA encoding structural proteins showing minor differences in the sequence.^49,50^ This fact hampers the rapid identification of SARS-CoV-2 variants with PCR or antigen-detecting rapid diagnostic tests.^51,52^ In this regard, SERS is advantageous to identify variants because it is based on the intrinsic fingerprint information of these proteins. As shown in Fig. 5a, the SERS spectrum of the SARS-CoV-2 N protein shows an obvious difference from that of the SARS-CoV S protein. This is not surprising because they show obvious differences in morphology as a result of the great variation in the sequence. We can also identify minor differences in SERS spectra of the SARS-CoV S protein and MERS-CoV S protein, two homologous proteins of coronavirus, benefiting from the high reliability and reproducibility of the proposed strategy. We then applied the strategy to characterize more challenging systems, *i.e.* S proteins from the wild type to its variants, as shown in Fig. 5b. As expected, it is extremely challenging to directly identify different S protein variants from their SERS spectra because of their high homology (with only *ca.* 15 mutation sites over 1206 amino acids). The PC scores illustrated in Fig. 5c reveal a clear separation of the spectra between the wild type and its variants, whereas variant proteins with high homology show an obvious overlap of clusters. We thus introduced AutoGluon,^53^ an automatic machine learning tool, and trained it to classify variant proteins from their SERS spectra. We achieved a good level of precision and recall (as summarized in Table S1,† both higher than 90%). To the best of our knowledge, we have demonstrated a cost-effective strategy for identifying protein variants by SERS, which has not been achieved before. The proposed strategy can be applied for bedside detection without any extraction and/or amplification and can be finished in 15 min, which shows its potential to handle a broad range of public health applications.

We have demonstrated a robust tool for dynamic characterization of the native protein, which allows us to investigate the ion effect on representative proteins with high reproducibility, reliability and sensitivity. It is interesting to note that the SERS spectrum of a specific protein varies with different research groups, whereas normal Raman spectra are the same. It cannot be merely attributed to different detection instruments used. For conventional methods (*e.g.*, AgIMNPs, AgIANPs, and Ag@IDCNPs), introducing different ions and exogenous species or performing by different persons also affects the final spectra of proteins, which hampers the cross-reference and cross-validation with the data from the literature. Different from other methods, we proposed a strategy to obtain a SERS signal of the native protein and introduced the correlation coefficient as a new criterion by referring to its normal Raman spectrum, which can be easily obtained in an ordinary laboratory. The high similarity between SERS and normal Raman spectra implies that the native state of proteins can be well retained during SERS measurement, which guarantees the high reproducibility of the method. As shown in Fig. 3d, the intensity and correlation coefficient reveal different trends during evaporation and the maxima of the correlation coefficient (native state) and intensity are located in different periods. Therefore, we cannot use intensity as a criterion (although it almost becomes a routine to use maximum intensity) to obtain the SERS spectra of the native protein. With the aid of the correlation coefficient, we can controllably and selectively detect the native protein with the maximal correlation coefficient, which makes dynamic tracking of the protein structure possible. It thus provides a platform for establishing a Raman and SERS database of proteins as well as a powerful tool for studying structure biology. Specifically, when the effect of detection conditions of traditional structure biological methods on the protein native structure is still unknown, our method could obtain SERS spectra of the native protein with high sensitivity and will be a competent tool for dynamic analysis of the native protein structure in a physiological environment.^54^

## Conclusions

To realize the label-free SERS characterization of the native proteins, we proposed iodide-coated 140 nm Au nanoparticles in combination with dynamic SERS characterization (D-AuIMNPs) to construct 3D physiological hotspots. By introducing the correlation coefficient in D-AuIMNPs, we demonstrated a quantitative and general strategy for dynamic characterization of the proteins in their native states. This strategy was successfully applied to monitor structural fluctuations and perturbation of other exogenous species on the native protein structure. It is advantageous to use the maximal value of the correlation coefficient instead of the intensity as the criterion to find the period to detect proteins in most native states. It prevents the loss of important intrinsic fingerprint information of the native protein with the intensity-based method. The strategy also enables the quantitative SERS investigation on the impact of ions at physiological concentrations on protein behavior, which agrees with the Hofmeister series. Furthermore, this strategy allows rapid and accurate identification of SARS-CoV-2 variant proteins, which has not been achieved before. Benefitting from the high reproducibility and dynamic characterization, this method provides a unique platform for establishing a SERS database of proteins, exhibits its potential for tracking dynamic structures of protein, and is promising to unveil the drug-protein interaction and study more complex systems.

## Data availability

The datasets supporting this article have been uploaded as part of the ESI material.†

## Author contributions

P.-S. W., H. M., X. W., and B. R. conceived the idea and designed the experiments. P.-S. W. finished the experiments, X.-H. X. and H. T. validated the results. H. M. and B. R. proposed the model and spectral interpretation. S. Y. performed FDTD simulations and X. L. conducted the data analysis and machine-learning coding. X.-H. P., Y.-F. B., Y. H., and M.-F. C. prepared the Au film. J. H. and G. L. provided the experimental resources. P.-S. W., H. M., and X. W. finished the draft of the manuscript. B. R. and G. L. reviewed and edited the final manuscript. H. M., X. W., and B. R. supervised the project. All authors participated in the result discussion and the manuscript preparation.

## Conflicts of interest

The authors declare no competing financial interest.

## Supplementary Material

SC-013-D2SC04775F-s001

## References

[cit1] Case Lindsay B., Zhang X., Ditlev Jonathon A., Rosen Michael K. (2019). Science.

[cit2] Schuth N., Mebs S., Huwald D., Wrzolek P., Schwalbe M., Hemschemeier A., Haumann M. (2017). Proc. Natl. Acad. Sci..

[cit3] Teo R. D., Migliore A., Beratan D. N. (2020). Chem. Sci..

[cit4] Donnelly A. E., Murphy G. S., Digianantonio K. M., Hecht M. H. (2018). Nat. Chem. Biol..

[cit5] Idso M. N., Akhade A. S., Arrieta-Ortiz M. L., Lai B. T., Srinivas V., Hopkins J. P., Gomes A. O., Subramanian N., Baliga N., Heath J. R. (2020). Chem. Sci..

[cit6] Suhre K., McCarthy M. I., Schwenk J. M. (2021). Nat. Rev. Genet..

[cit7] Tamara S., den Boer M. A., Heck A. J. R. (2022). Chem. Rev..

[cit8] Zhou M., Lantz C., Brown K. A., Ge Y., Paša-Tolić L., Loo J. A., Lermyte F. (2020). Chem. Sci..

[cit9] Wu C., Garden P. M., Walt D. R. (2020). J. Am. Chem. Soc..

[cit10] Chim N., Meza R. A., Trinh A. M., Yang K., Chaput J. C. (2021). Nat. Commun..

[cit11] Maeki M., Ito S., Takeda R., Ueno G., Ishida A., Tani H., Yamamoto M., Tokeshi M. (2020). Chem. Sci..

[cit12] Stiller J. B., Otten R., Häussinger D., Rieder P. S., Theobald D. L., Kern D. (2022). Nature.

[cit13] Kim J. G., Kim T. W., Kim J., Ihee H. (2015). Acc. Chem. Res..

[cit14] Christopher J. A., Stadler C., Martin C. E., Morgenstern M., Pan Y., Betsinger C. N., Rattray D. G., Mahdessian D., Gingras A.-C., Warscheid B., Lehtiö J., Cristea I. M., Foster L. J., Emili A., Lilley K. S. (2021). Nat. Rev. Methods Primers.

[cit15] Nie S., Emory S. R. (1997). Science.

[cit16] Zong C., Xu M., Xu L.-J., Wei T., Ma X., Zheng X.-S., Hu R., Ren B. (2018). Chem. Rev..

[cit17] Cotton T. M., Schultz S. G., Vanduyne R. P. (1980). J. Am. Chem. Soc..

[cit18] Buhrke D., Hildebrandt P. (2020). Chem. Rev..

[cit19] Xu H., Bjerneld E. J., Käll M., Börjesson L. (1999). Phys. Rev. Lett..

[cit20] Xu H., Aizpurua J., Käll M., Apell P. (2000). Phys.
Rev. E.

[cit21] Ma H., Tang X., Liu Y., Han X. X., He C., Lu H., Zhao B. (2019). Anal. Chem..

[cit22] Han X. X., Huang G. G., Zhao B., Ozaki Y. (2009). Anal. Chem..

[cit23] Guerrini L., Krpetić Ž., van Lierop D., Alvarez-Puebla R. A., Graham D. (2015). Angew. Chem., Int. Ed..

[cit24] Xu L.-J., Zong C., Zheng X.-S., Hu P., Feng J.-M., Ren B. (2014). Anal. Chem..

[cit25] Bao Y., Li Y., Ling L., Xiang X., Han X., Zhao B., Guo X. (2020). Anal. Chem..

[cit26] Li D., Zhang Z., Wang X., Wang Y., Gao X., Li Y. (2022). Biosens. Bioelectron..

[cit27] Li Y., Han X., Zhou S., Yan Y., Xiang X., Zhao B., Guo X. (2018). J. Phys. Chem. Lett..

[cit28] Singh S., Agarwal A., Avni A., Mukhopadhyay S. (2021). J. Phys. Chem. Lett..

[cit29] Wrinch D. (1947). Science.

[cit30] Henzler-Wildman K., Kern D. (2007). Nature.

[cit31] Yang L., Li P., Liu H., Tang X., Liu J. (2015). Chem. Soc. Rev..

[cit32] Yang L., Liu H., Wang J., Zhou F., Tian Z., Liu J. (2011). Chem. Commun..

[cit33] Fang P.-P., Li J.-F., Yang Z.-L., Li L.-M., Ren B., Tian Z.-Q. (2008). J. Raman Spectrosc..

[cit34] Ye S., Zhong K., Zhang J., Hu W., Hirst J. D., Zhang G., Mukamel S., Jiang J. (2020). J. Am. Chem. Soc..

[cit35] Grant A. M., Krecker M. C., Gupta M. K., Dennis P. B., Crosby M. G., Tsukruk V. V. (2020). ACS Biomater. Sci. Eng..

[cit36] Pegram L. M., Wendorff T., Erdmann R., Shkel I., Bellissimo D., Felitsky D. J., Record M. T. (2010). Proc. Natl. Acad. Sci. U. S. A..

[cit37] Metrick M. A., do Carmo Ferreira N., Saijo E., Hughson A. G., Kraus A., Orrú C., Miller M. W., Zanusso G., Ghetti B., Vendruscolo M., Caughey B. (2019). Proc. Natl. Acad. Sci. U. S. A..

[cit38] Lo Nostro P., Ninham B. W. (2012). Chem. Rev..

[cit39] Zhang Y., Cremer P. S. (2006). Curr. Opin. Chem. Biol..

[cit40] Jordan J. H., Gibb C. L. D., Wishard A., Pham T., Gibb B. C. (2018). J. Am. Chem. Soc..

[cit41] Constantinescu D., Weingärtner H., Herrmann C. (2007). Angew. Chem., Int. Ed..

[cit42] He X., Zhang K., Liu Y., Wu F., Yu P., Mao L. (2018). Angew. Chem., Int. Ed..

[cit43] Wei W., Chen X., Wang X. (2022). Small.

[cit44] Ashraf H., Guo Y., Wang N., Pang S., Zhang Y.-H. (2021). J. Phys. Chem. A.

[cit45] Cacace M. G., Landau E. M., Ramsden J. J. (1997). Q. Rev. Biophys..

[cit46] Singh J., Rahman S. A., Ehtesham N. Z., Hira S., Hasnain S. E. (2021). Nat. Med..

[cit47] Baric R. S. (2020). N. Engl. J. Med..

[cit48] Cao Y., Yisimayi A., Jian F., Song W., Xiao T., Wang L., Du S., Wang J., Li Q., Chen X., Yu Y., Wang P., Zhang Z., Liu P., An R., Hao X., Wang Y., Wang J., Feng R., Sun H., Zhao L., Zhang W., Zhao D., Zheng J., Yu L., Li C., Zhang N., Wang R., Niu X., Yang S., Song X., Chai Y., Hu Y., Shi Y., Zheng L., Li Z., Gu Q., Shao F., Huang W., Jin R., Shen Z., Wang Y., Wang X., Xiao J., Xie X. S. (2022). Nature.

[cit49] Tao K., Tzou P. L., Nouhin J., Gupta R. K., de Oliveira T., Kosakovsky Pond S. L., Fera D., Shafer R. W. (2021). Nat. Rev. Genet..

[cit50] Ye G., Liu B., Li F. (2022). Nat. Commun..

[cit51] Crozier A., Rajan S., Buchan I., McKee M. (2021). BMJ.

[cit52] Migueres M., Lhomme S., Trémeaux P., Dimeglio C., Ranger N., Latour J., Dubois M., Nicot F., Miedouge M., Mansuy J. M., Izopet J. (2021). J. Clin. Virol..

[cit53] Erickson N. , Mueller J., Shirkov A., Zhang H., Larroy P., Li M. and Smola A., arXiv, 2003, preprint, arXiv:2003.06505, 10.48550/arXiv.2003.06505

[cit54] Ourmazd A., Moffat K., Lattman E. E. (2022). Nat. Methods.

