# Supplementary material for: Correlation coefficient-directed label-free characterization of native proteins by surface-enhanced Raman spectroscopy
Source: Chem Sci. 2022 Oct 31;13(46):13829–35. doi: 10.1039/d2sc04775f (PMC9710310; doi:10.1039/d2sc04775f)
Supplement: SC-013-D2SC04775F-s001 [file SC-013-D2SC04775F-s001.pdf]

Electronic Supplementary Material (ESI) for Chemical Science.
This journal is © The Royal Society of Chemistry 2022

Supporting information:

Correlation Coefficient-Directed Label-Free Characterization of Native Proteins by Surface-Enhanced Raman Spectroscopy

Ping-Shi Wang,[a] Hao Ma,*[a] Sen Yan,[a] Xinyu Lu,[a] Hui Tang,[a] Xiao-Han Xi,[a] Xiao-Hui Peng,[a] Yajun Huang,[a] Yi-Fan Bao,[a] Mao-Feng Cao,[a] Huimeng Wang,[a] Jinglin Huang,[b] Guokun Liu,[c] Xiang Wang,*[a] Bin Ren*[a]

[a]State Key Laboratory of Physical Chemistry of Solid Surfaces, Collaborative Innovation Center of Chemistry for Energy Materials (i-ChEM), Innovation Laboratory for Sciences and Technologies of Energy Materials of Fujian Province (IKKEM), Department of Chemistry, College of Chemistry and Chemical Engineering, Xiamen University, Xiamen 361005, China

[b]Laser Fusion Research Center, China Academy of Engineering Physics, Mianyang 621900, China

[c]State Key Laboratory of Marine Environmental Science, College of the Environment and Ecology, Xiamen University, Xiamen 361005, China

Email: oaham@xmu.edu.cn (H.M.); wangxiang@xmu.edu.cn (X.W.); bren@xmu.edu.cn (B.R.)

Table of Contents

Supplementary experimental details.

Computational details.

Supplementary Figures:

Figure S1. Normal Raman spectra of the saturated aqueous solution of lysozyme, and SERS spectrum of lysozyme with AuIMNPs on hydrophobic Au film and Si wafer.

Figure S2. FDTD simulation of nanoparticles with different sizes.

Figure S3. SEM images of AuIMNPs and AgNPs.

Figure S4. Normal Raman and SERS spectra in different environments: a. BSA (pI=4.7), b. Cyt C (pI=10.7), c. EA (pI=4.5).

Figure S5. Time course of dynamic characterization lysozyme and its corresponding SERS spectra.

Figure S6. Ions effect of different cations and anions on lysozyme at different salt concentrations.

Figure S7. Ions effect of different cations and anions on BSA at different salt concentrations.

Figure S8. Ions effect of different cations and anions on Cyt C at different salt concentrations.

Figure S9. Schematic illustration of the proposed strategy for identification of variant proteins of SARS-CoV-2.

Figure S10. SERS spectra of SARS-CoV-2 S protein.

Table S1. Performance of AutoGluon for identification of variant proteins.

References.

1. Experimental Details

1.1 Chemicals

Lysozyme and egg ovalbumin (EA) were purchased from Beijing Solarbio Co., Ltd. bovine serum albumin (BSA) was obtained from Aladdin. Oxidized cytochrome c was purchased from Perfemiker Co., Ltd. Recombinant wild-type S and N proteins of SARS-CoV-2, S protein of SARS-CoV and MERS-CoV, and 3 recombinant variant S proteins of SARS-CoV-2 were purchased from Beijing Yiqiao Shenzhou Technology Co., Ltd. Three variant proteins of SARS-CoV-2 are South Africa B.1.351 lineage (L18F, D80A, D215G, LAL242-244 miss, R246I, K417N, E484K, A701V), South Africa B.1.351 lineage (D80A, K417N, E484K, N501Y, D614G, A701V), Brazil P. 1 (L18F, T20N, P26S, D138Y, R190S, K417T, E484K, N501Y, D6517Y, T197F, V117F). Ultrafiltration tubes with a molecular weight cutoff of 30 and 100 kDa were purchased from Merck Millipore (for N protein and S protein respectively). High-purity water (Milli-Q, 18.2 MΩ cm) was utilized throughout the research.

1.2 Preparation of atomically smooth gold films and hydrophobic Si wafer

Atomically smooth gold films were fabricated based on the template stripped method, where a clean Si(111) single crystal wafer was used as a template. 200 nm Au film was deposited by electron beam evaporation (Temescal, FC-200) at a rate of 0.35 nm/s and a pressure of 5×10^{-7} Torr. Then UV glue (Norland Optical Adhesive, NOA81) was used to adhere to a small glass slide on top of the Au film. After 15 minutes of UV illumination to cure the glue, the smooth gold film surface can be obtained by taking off the slide. Hydrophobic Si wafers were obtained by the 40% HF solution etching.

1.3 Preparation of Iodide-Modified Au NPs (Au IMNPs)

Uniform and regular spherical 140 nm AuNPs were prepared by a multi-step synthesis method in our previous work.[1] AuIMNPs were prepared similarly to our previous method. Briefly, 2 mL of gold colloids were centrifuged (3000 rpm, 4 min) and the supernatant was removed. Then 20 μL of 10 mM potassium iodide was added to 1 mL of gold colloid and sonicated at 25 °C for 20 min. After centrifuging the mix, the supernatant was removed. The process was performed at least three times to confirm

that impurities on AuNPs have been completely removed.

1.4 SERS characterization and analysis

A mixed droplet of 2 μL of protein solution and 2 μL of Au IMNPs was placed on the surface of the gold film and dried in vacuum for 2 min. The SER spectrum collection starts when the droplet transforms into a liquid film. Normal Raman and SER spectra were conducted by a confocal Raman system (Nanophoton, Japan) equipped with semiconductor laser (785 nm). The laser powers for SERS and normal Raman measurements are 8 and 30 mW, respectively. The typical exposure time for each measurement applied in this work was 3 s with one-time accumulation unless otherwise stated.

For the detection of SARS-CoV-2 variant proteins, normal Raman and SERS spectra were obtained by a fiber-coupled Raman system (Renishaw Virsa, UK) using a 785 nm laser as excitation light. The laser powers for SERS and normal Raman measurements are about 1 and 30 mW, respectively.

2. Computational details:

The electromagnetic field distributions of the AuNPs were calculated by using the 3D finite-difference time-domain (FDTD) simulation (Lumerical Solutions, Canada). In the simulations, AuNPs with a diameter of 140 nm were placed in the cube with perfectly matched layers (PML). PML conditions were adopted surrounding the simulation domain to avoid unnecessary boundary reflections. A linearly polarized plane wave light was used to excite the dimer at normal incidence. The dielectric function of gold was extracted from Johnson and Christy's data.[2] In order to obtain the electromagnetic field distribution in a narrow gap, the maximum unit cell was 1 nm × 1 nm × 1 nm.

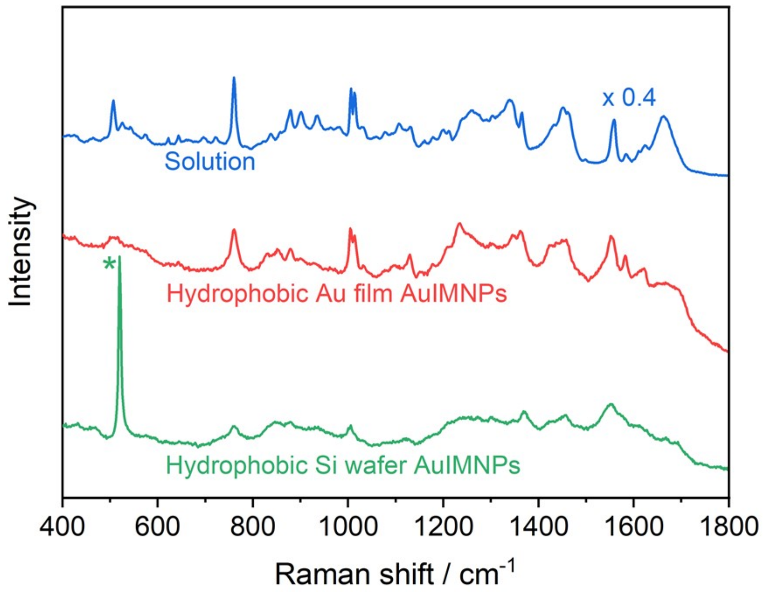

Figure S1. Normal Raman spectra of the saturated aqueous solution of lysozyme, and SERS spectrum of lysozyme with AuIMNPs on hydrophobic Au film and Si wafer. * Peak from silicon.

As shown in Figure S1, we conducted the same experiments on the hydrophobic Si wafer, giving 1.64 times lower SERS intensity of lysozyme than that on the Au film. The spectra obtained on Au film give clearer peaks than that on hydrophobic Si wafer. It is deduced that the high reflection of the Au film would contribute more enhancement of the SERS signal. More importantly, there may exist a synergistic enhancement as a result of the coupling between nanoparticles, and nanoparticles and Au film (so called gap-mode).

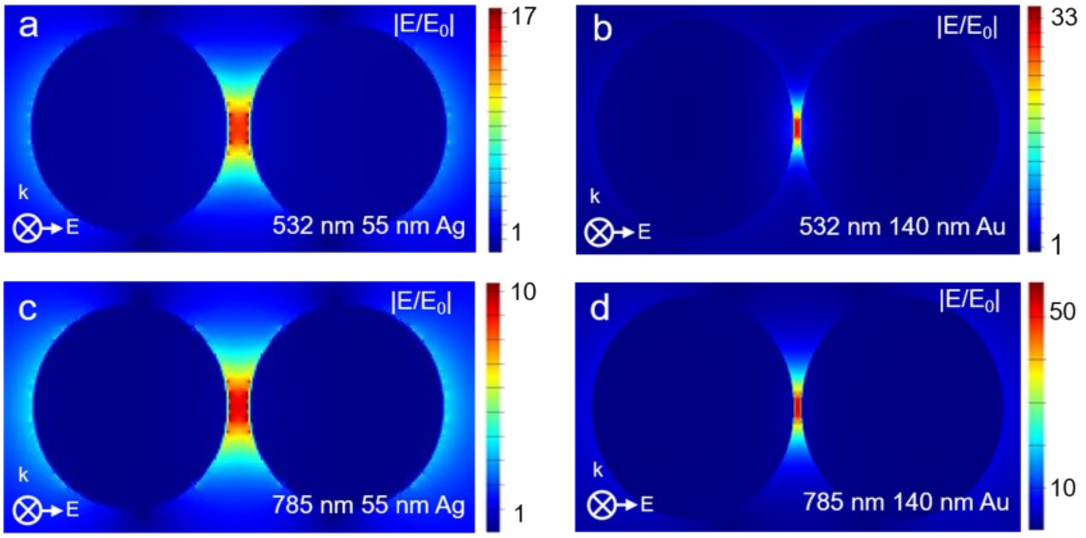

Figure S2. FDTD simulation of nanoparticles (NPs) with different size under different incident light (Gap distance = 5 nm).

On the one hand, as can be seen in Figure S2, the 55 nm AgNPs have stronger electromagnetic field under 532 nm than 785 nm, whereas 140 nm AuNPs perform better under 785 nm than 532 nm. As a result, AgIMNPs provide higher enhancement with incident light of 532 nm, and AuIMNPs have better performance under 785 nm. On the other hand, the gap can be regarded as a quasi-uniform electromagnetic field owing to the small gradient, as shown in Figure S2.

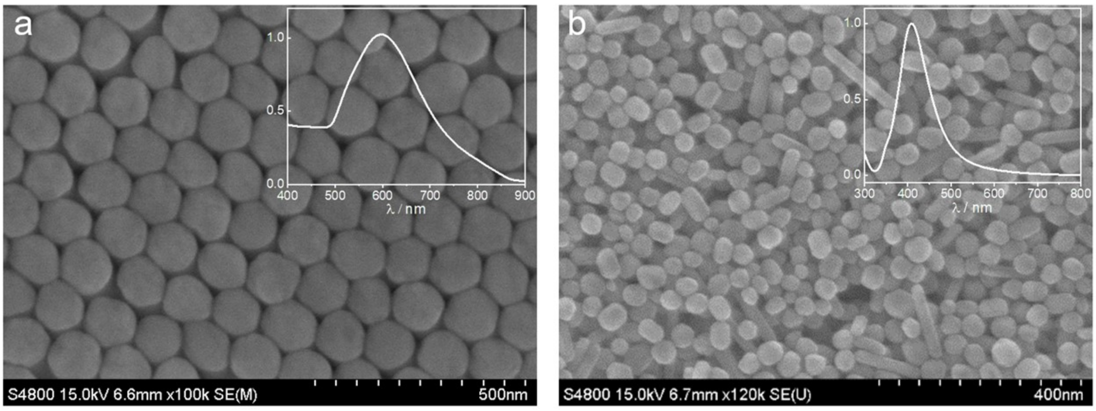

Figure S3. SEM images of 140 nm AuIMNPs used in the manuscript and normally used 55 nm AgIMNPs used in the literature. The inset spectra are the UV-Vis spectra of corresponding nanoparticles. It can be found that, the AgIMNPs are not as uniform as AuIMNPs, which would result in bad reproducibility.

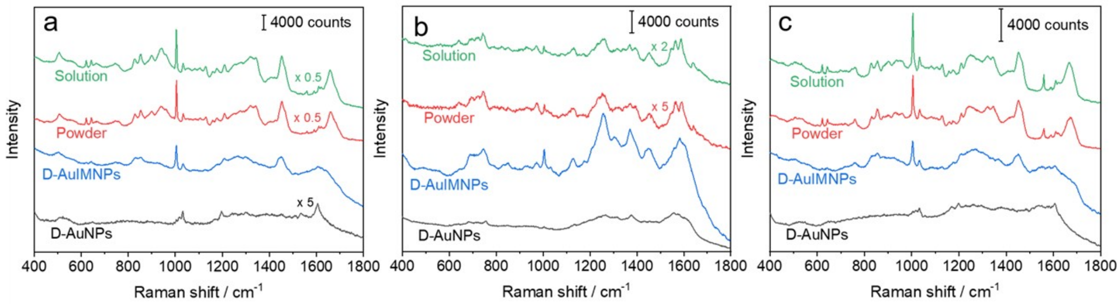

Figure S4. Normal Raman and SERS spectra in different environments: a. BSA (pI=4.7), b. Cyt C (pI=10.7), c. EA (pI=4.5).

Three proteins in Figure S4 have different isoelectronic points (pI) from acid to base. The *R* decreases from normal Raman spectra of solution and solid, to SERS spectra on D-AuIMNPs to AuNPs, showing a trend similar to that in the lysozyme system. Therefore, the *R* can be applied to reflect the native degree from the SERS spectra of a variety of proteins, no matter acidic or alkaline, with or without cofactors. For Cyt C, although it also follows the same trend, the *R* value is close to each other under different detection conditions, as the SERS signal is mainly contributed by the porphyrin moiety encapsulated inside the backbone chain, which is insensitive to the detection environment. Moreover, our method can reliably reproduce the SERS spectra without adding any aggregation agent with high *R* of 0.9, indicating that our method can preserve the native state of proteins. This method allows us to use SERS for analyzing the minute change of the protein structure under the influence of other factors.

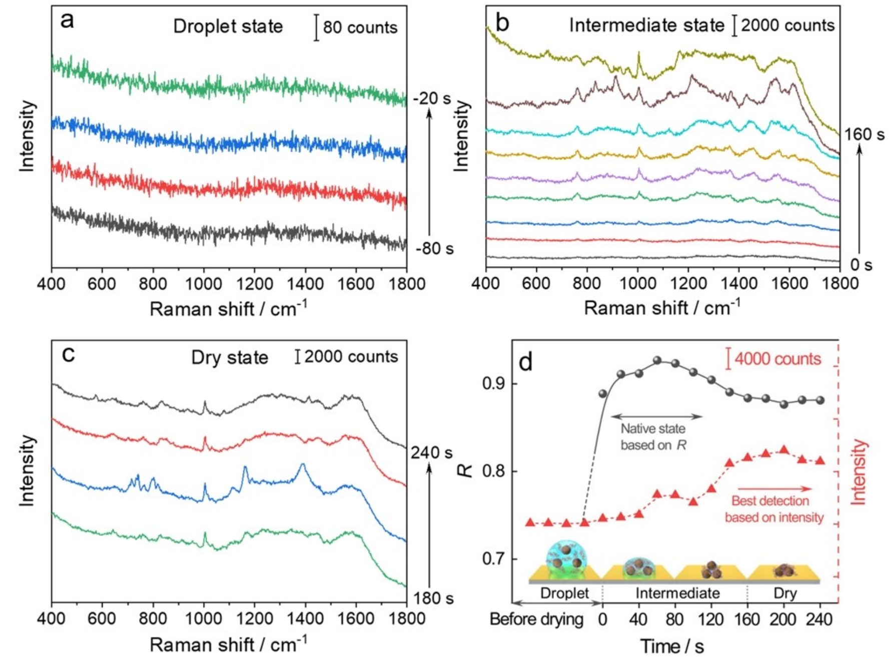

Figure S5. Time course of dynamic characterization of lysozyme and its corresponding SERS spectra in: a. Droplet state, b. Intermediate state, c. Dry state. d. Correlation coefficients (*R*, solid line) and changes of the intensity of the peak at 1004 cm^{-1} (dotted line) during solvent evaporation.

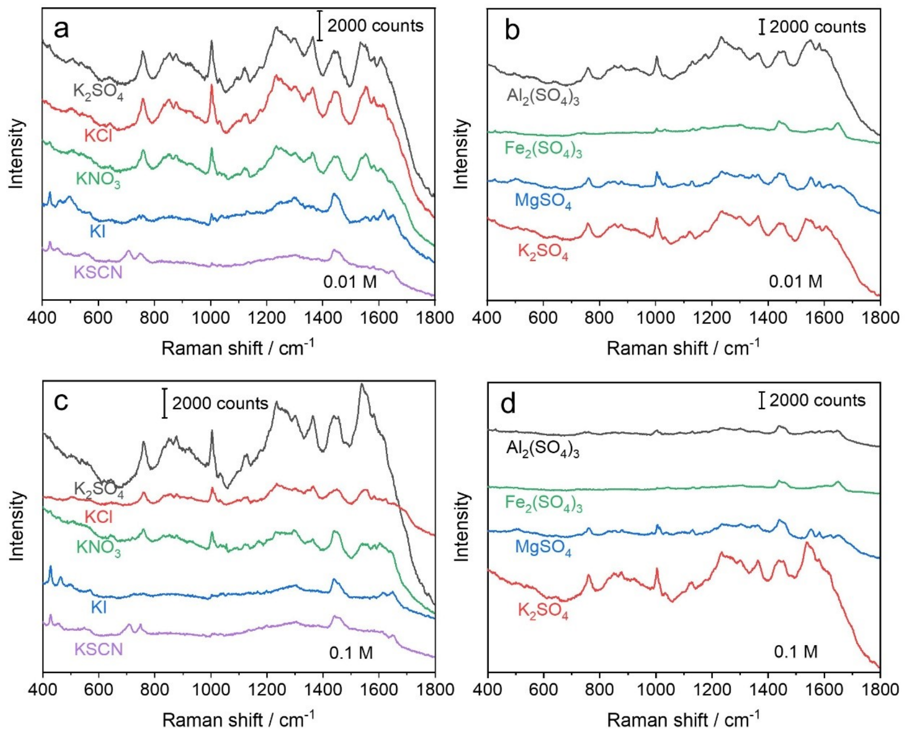

Figure S6. Ions effect of different cations and anions on lysozyme at different salt concentrations: a. Different cations at 0.01 M, b. Different anions at 0.01 M, c. Different cations at 0.1 M, d. Different anions at 0.1 M.

It should be noted that we can obtain the signals of ions at high concentration, but the influence on the results of correlation coefficient is neglectable. Moreover, the concentrations of protein and ions are increasing during evaporation. We detected each sample in the very beginning of intermediate state to minimize the change of the concentrations.

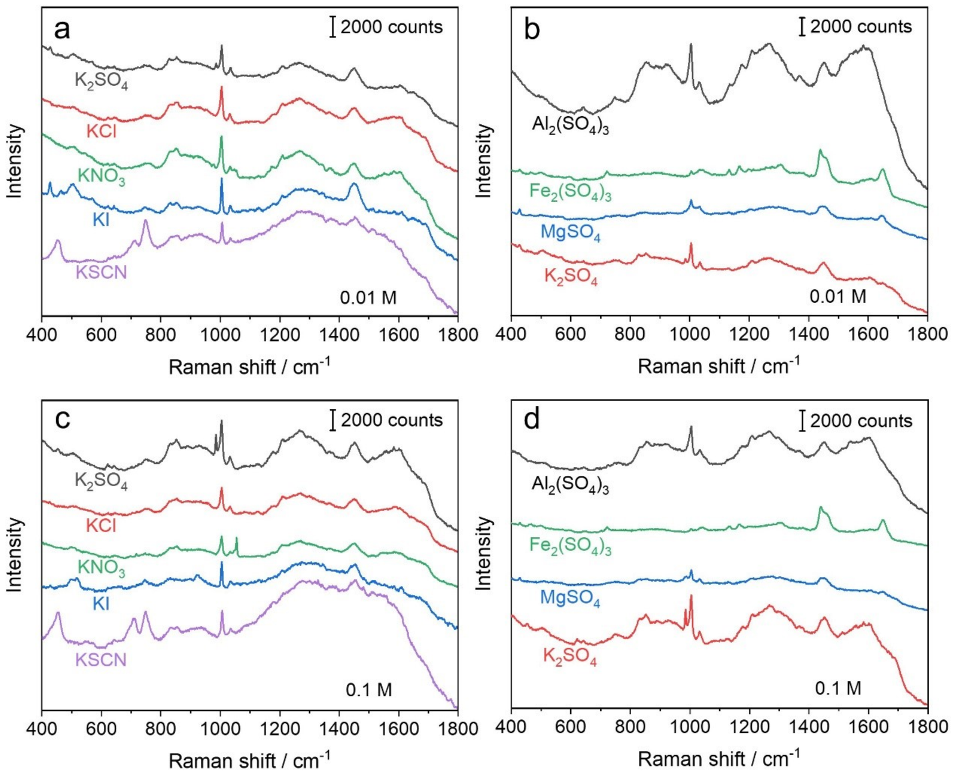

Figure S7. Ions effect of different cations and anions on BSA at different salt concentrations: a. Different cations at 0.01 M, b. Different anions at 0.01 M, c. Different cations at 0.1 M, d. Different anions at 0.1 M.

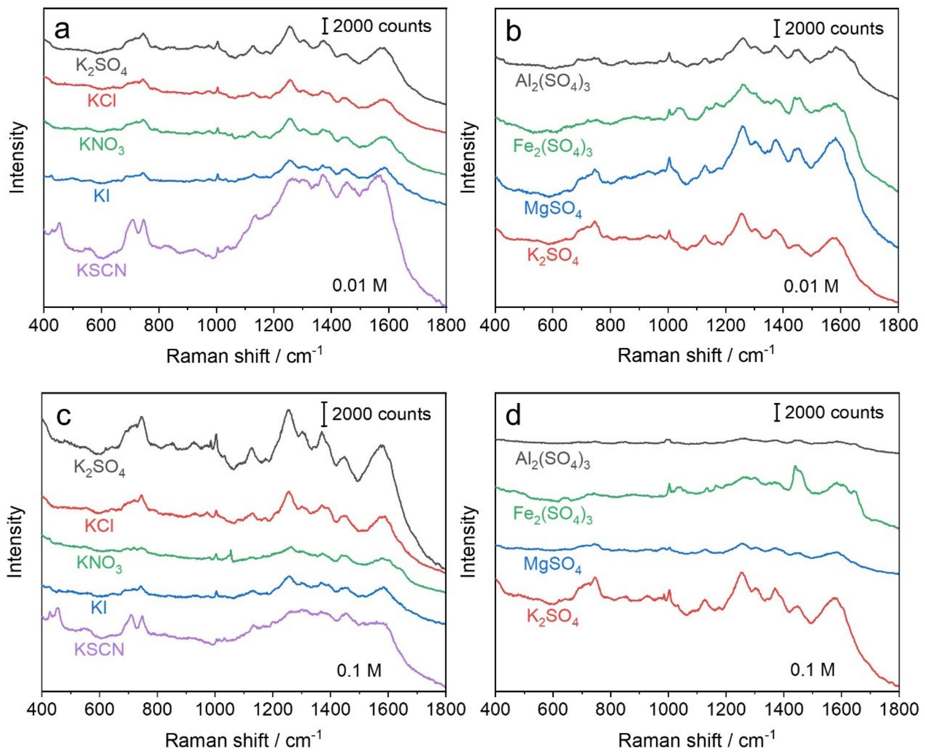

Figure S8. Ions effect of different cations and anions on Cyt C at different salt concentrations: a. Different cations at 0.01 M, b. Different anions at 0.01 M, c. Different cations at 0.1 M, d. Different anions at 0.1 M.

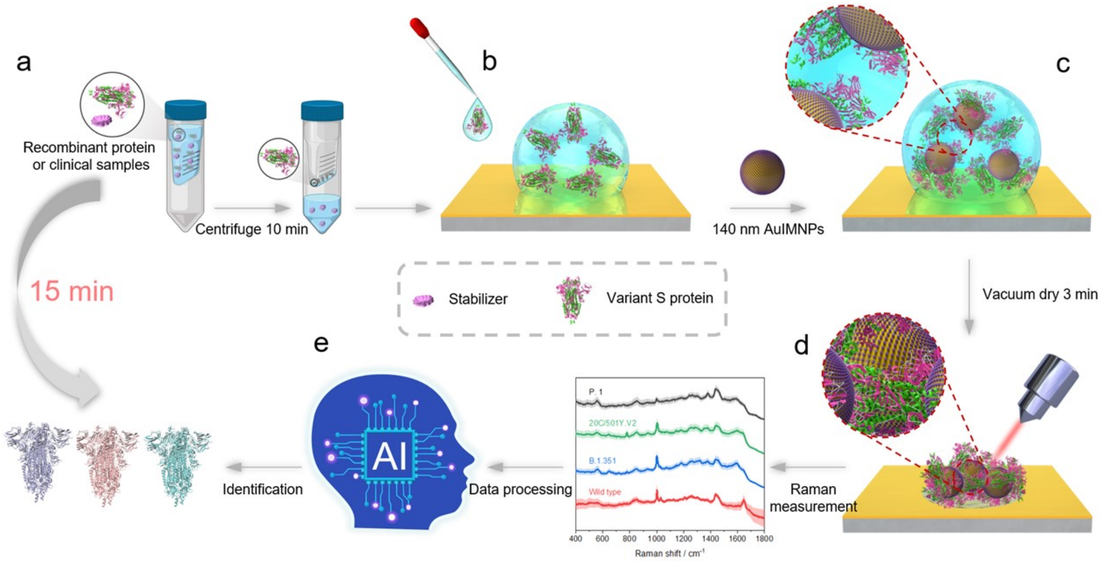

Figure S9. Schematic illustration of the proposed strategy for identification of variant proteins of SARS-CoV-2.

The protocol for identifying variant proteins of SARS-CoV-2 is illustrated in Figure S9. We first utilized ultrafiltration tubes to purify and preconcentrate the samples (solution of recombinant variant proteins as the model) for removing small protective agents. Then, 1.5 μL of S protein solution was dropped on the gold film, where the hydrophobic surface kept the solution as a droplet (Figure S9b). To the droplet of the solution, 1.5 μL of 140 nm AuIMNPs was then added to form a mixture (Figure S9c). At last, the SERS detection of variants was performed right after the intermediate state (Figure S9d, See Figure S10 for the spectra). With aid of machine learning (ML), we can identify different variant proteins. The performance of ML method used is summarized in Table S1.

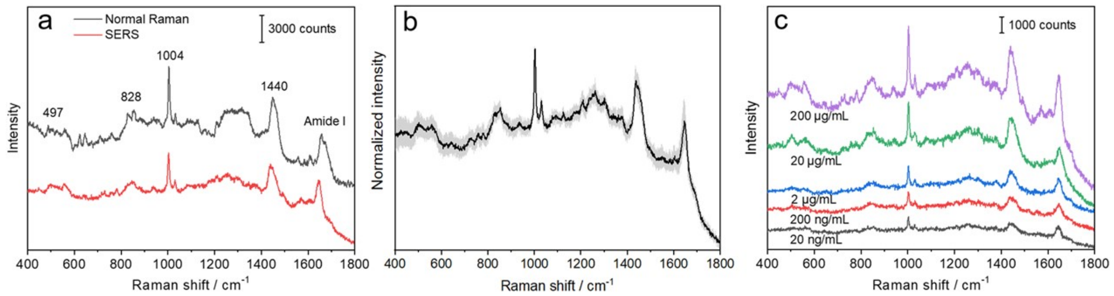

Figure S10. SERS spectra of SARS-CoV-2 S protein. a. SERS spectrum of SARS-CoV-2 S protein in comparison with its normal Raman spectrum. b. The averaged spectrum and deviations of spectra (indicated by gray shadow) obtained in 15 different regions on the 200 μg/mL SARS-CoV-2 S protein sample. c. SERS signals of the SARS-CoV-2 S protein at different concentrations.

Table S1. Performance of AutoGluon for identification of variant proteins.

**Sample**	**Accuracy**	**Precision**	**Recall**	**F_1 score**
Wild type	0.92	1.00	0.92	0.96
B.1.351	1.00	0.91	1.00	0.96
20C/501.Y.V 2	0.91	1.00	0.91	0.95
P.1	1.00	1.00	1.00	1.00
Total	0.96	0.96	0.96	0.96

The performance of AutoGluon for identification of variant proteins is summarized in Table S1. The accuracy is the proportion of samples that are correctly classified. The precision is the ratio of true positive samples that are classified as positive. The recall is the ratio of positive samples that are correctly classified as positive. The F_1 score is the harmonic mean of precision and recall. The support is the proportion of samples of a certain class in total data set. The definition of different evaluation metrics are listed from Equation 1 to Equation 5:

$$Accuracy = \frac{TP + TN}{TP + TN + FP + FN} \qquad (Eq\ 1)$$

$$Precision = \frac{TP}{TP + FP} \qquad (Eq\ 2)$$

$$Recall = \frac{TP}{TP + FN} \qquad (Eq\ 3)$$

$$F_1\ score = 2\frac{precision \cdot recall}{precision + recall} \qquad (Eq\ 4)$$

$$Support = \frac{n_{class}}{n_{total}} \qquad (Eq\ 5)$$

where TP, FP, TN, FN, n_{class} and n_{total} represent true positive, false positive, true negative, false negative, the number of samples of a certain class and size of total data set, respectively. In binary classification, the samples were labelled either positive or

negative. If the prediction and actual value are both positive, it is TP; if the prediction and actual value are both negative, it is TN; if the prediction value is positive and the actual value is negative, it is FP; and if the prediction value is negative while the actual value is positive, it is FN.

References:

1. P.-P. Fang, J.-F. Li, Z.-L. Yang, L.-M. Li, B. Ren and Z.-Q. Tian, *J. Raman Spectrosc.*, 2008, **39**, 1679-1687.
2. P. B. Johnson and R. W. Christy, *Phys. Rev. B*, 1972, **6**, 4370-4379.
